# Nuclear RNA cap-chaperones eIF4E and NCBP2 govern distinct fates for 1000s of mRNAs uncovering an unexpected regulatory point in gene expression

**DOI:** 10.1101/2025.07.25.666897

**Published:** 2025-07-31

**Authors:** Jean-Clement Mars, Caleb M. Embree, Biljana-Culjkovic-Kraljacic, Aidan W.B. Carlile, Patrick Gendron, Katherine L.B. Borden

**Affiliations:** 1 Department of Pathology and Cell Biology, Institute for Research in Immunology and Cancer, Université de Montréal, Québec, Canada.; 2 Department of Pharmacology and Robert H. Lurie Cancer Centre, Northwestern University, Chicago, Illinois, USA

## Abstract

mRNA processing constitutes a series of steps in the nucleus to generate mature mRNAs that can be translated into protein. This relies on the methyl-7-guanosine (m^7^G) “cap” on the 5’end of mRNAs which is bound by the nuclear cap-binding protein NCBP2 with its cofactor NCBP1. The NCBP1/2 complex chaperones capped mRNA through these processing steps. NCBP2 is considered the sole nuclear cap-binding factor and thus its cap-chaperone role is thought to be a constitutive, housekeeping activity. However, another cap-binding protein, the eukaryotic translation initiation factor eIF4E, is also found in the nucleus. Two cap-binding factors co-existing in the nucleus intimate an undiscovered regulatory point in gene expression or, alternatively, redundancy to ensure gene expression fidelity. Consistent with the former possibility, eIF4E and NCBP2 drove distinct gene expression, transcriptomic, and splicing signatures impacting ~2500 transcripts involved in distinct biological programmes with only ~360 transcripts in common and of these only 79 common splicing events. Thus, each cap-chaperone designates distinct mRNA populations for specific processing revealing a new step in gene expression. We denote this mRNA specification of cap-chaperones (SOCCS). We uncovered multiple molecular mechanisms that contribute to SOCCS: distinct spatial localization of eIF4E and NCBP2 within the nucleus, identification of sequence motifs within targeted mRNAs segregated by eIF4E or NCBP2 sensitivity, distinct protein partners for these cap-chaperones and differential impacts on the production of key spliceosome components e.g. U2AF1, PRP31, SF3B1 and SNRNP200 indicative of distinct transcriptomic landscapes produced by eIF4E or NCBP2 overexpression. In all, the realization that multiple cap-binding proteins coexist in the nucleus led us to identify an unexpected gene-expression regulatory point which engaged distinct biological programmes.

## Introduction

**Figure 1:**
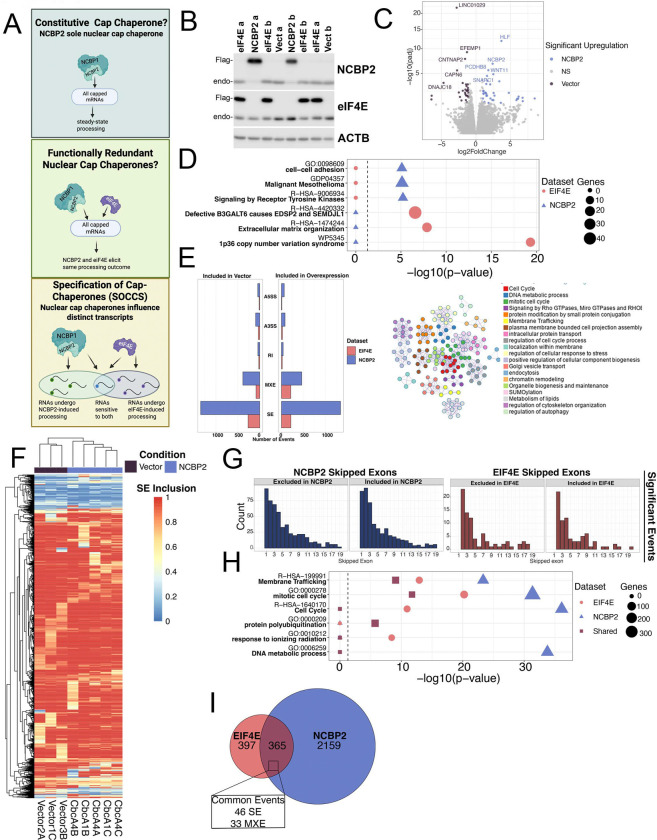
EIF4E and NCBP2 alter the splicing of distinct targets. **A)** The potential models of cap chaperones interacting with nuclear mRNA. Created with https://BioRender.com **B)** Western blot depicting the expression levels of EIF4E and NCBP2 overexpression in U2OS cells from two clones (a and b). **C)** Volcano plot depicting the genes that are significantly upregulated (blue) or downregulated (purple) upon NCBP2 overexpression in U2OS cells. **D)** The top gene ontology terms of genes undergoing differential expression in EIF4E (red circles) or NCBP2 (blue triangles). Size of the point indicates the number of genes. Dashed line indicates p-value = 0.05. **E)** *Left* Bar graph illustrating the number of alternative splicing events in EIF4E (red) and NCBP2 (blue). *Right* Network analysis of enriched gene ontology terms from genes with skipped exon (SE) and mutually exclusive exon (MXE) alternative splicing events in NCBP2 overexpression. **F)** Heatmap of significantly changing skipped exon events showing inclusion (red) or exclusion (blue) of the exon in each sample. **G)** Position of significantly changing skipped exons in following overexpression of NCBP2 (blue, left) or EIF4E (red, right). **H)** Top gene ontology terms of genes undergoing alternative splicing in NCBP2 only (triangle, blue), EIF4E only (circle, red), or both (square, purple) overexpression. Size of the point indicates the number of genes. Dashed line indicates p-value = 0.05. **I)** Euler plot comparing the genes that have significantly changing alternative splicing in eIF4E (red) or NCBP2 (blue) overexpression. The number of skipped exons (SE) and mutually exclusive exon (MXE) events that are changing in both overexpression are indicated.

Gene expression encompasses all the processes involved in converting genetic information into functional protein. In eukaryotes, an essential step in this process is the addition of the methyl-7-guanosine (m^7^G) cap on the 5’ end of coding transcripts^1–3^. Capping of mRNA is conserved in animals, plants and fungi^1–3^. Caps are added to coding mRNAs at or near sites of transcription^1–4^. Ingrained in the central dogma of gene expression is the necessity for m^7^G-capped transcripts to be chaperoned through processing steps and subsequently escorted to the nuclear pore for export to the cytoplasm for translation^5–7^. The major purpose of the cap-chaperone is to mediate interactions between mRNAs and the appropriate processing machineries to enable maturation^6^. To date, this chaperone role was solely attributed to the nuclear cap-binding protein NCBP2 (CBP20) and its co-factor NCBP1 (CBP80) (Figure 1A, top panel). NCBP1 binds directly to NCBP2 and other RNA-binding proteins to direct mRNA fate^6,8,9^. Like the cap, NCBP2 and NCBP1 are evolutionarily conserved across kingdoms^5,6,10^. In this model, NCBP2/NCBP1 acts in processing and nuclear export with an exchange of mRNAs in the cytoplasm with the major cytoplasmic cap-binding protein eIF4E which engages transcripts in steady-state translation^11,12^.

Much that underpins the above model arises from the misconception that NCBP2 is the only cap-binding factor in the nucleus (Figure 1A). Indeed, eIF4E is found in the nucleus and cytoplasm in plants, animals and fungi^10,13,14^. This evolutionary conservation suggests a central role in capped mRNA metabolism in the nucleus. Consistently, eIF4E binds to many, ~3000 mRNAs, but not all mRNAs in the nucleus^15^. These observations suggest selectivity, whereby the cap is not sufficient to imbue all nuclear mRNAs with affinity for eIF4E^1^. For example, the eIF4E-sensitivity element (4ESE) is an element in the 3’UTR which sensitize mRNAs to eIF4E-dependent nuclear export through recruitment of co-factors to engage the nuclear pore complex^15–18^. In the 2000s, the role of eIF4E in the nucleus was thought to be limited to mRNA export of 4ESE-containing RNAs with no role in processing^16,19–24^. However, recent studies revealed that nuclear eIF4E interacted with the capping, CPA, and splicing machineries as well as some unspliced mRNAs and their spliced products^16,20,21,24–27^. As in the cytoplasm, nuclear eIF4E interacts with mRNAs in a cap-dependent manner as seen by studies using m^7^G cap competition and the eIF4E cap-binding mutant W56A^16,22–24,28^. Additionally, eIF4E influenced the production of the splicing, capping, and CPA machinery through increased mRNA export and in some cases enhanced translation efficiency of the transcripts encoding the relevant machinery^16,19–24^. This strongly suggests that eIF4E plays roles beyond mRNA export in the nucleus including influencing processing both directly through interactions with the machinery and indirectly through altering the production of key processing factors. Given eIF4E and NCBP2 co-exist in the nuclei of yeast, animal and plant cells^9,10,13,14,22,29^, it seems likely that there is an evolutionarily conserved purpose for this coexistence.

Given these findings, we re-examined the traditional NCBP2-restricted nuclear cap-chaperone model for mRNA processing (Figure 1A, top panel). We investigated whether the presence of both eIF4E and NCBP2 is a manifestation of functional redundancy in the eukaryotic nucleus to ensure gene expression fidelity, or if it constitutes an undiscovered step in the control of gene expression targeting selected subgroups of mRNAs (Figure 1A middle and bottom panels). Supporting the latter possibility, we observed that NCBP2- and eIF4E-overexpression elicited distinct gene expression profiles with little overlap. This supports a model whereby selected cap-chaperones designate certain mRNAs’ fate, a process we refer to as mRNA Specification of Cap Chaperones (SOCCS). SOCCS constitutes a previously unknown step in the control of gene expression for thousands of mRNAs and represents a significant shift from the current NCBP2 only cap-chaperone paradigm.

## Results

### eIF4E and NCBP2 elicit divergent transcriptomic and splicing signatures

We investigated whether the co-existence of nuclear eIF4E and NCBP2 reflected redundancy in processing to protect the fidelity of gene expression or if mRNAs were specified for a particular cap-chaperone protein to govern their fates. We used human osteosarcoma cells (U2OS) which are characterized by nuclear and cytoplasmic eIF4E and have normal eIF4E levels compared to healthy volunteers^25^. These cells also have nuclear NCBP2 and NCBP1 (see below). We first compared the transcriptional signatures of eIF4E and NCBP2 overexpressing cells. For this purpose, first examined whether eIF4E impacted steady-state transcription using RNA-Seq data comparing stably overexpressing 2FLAG-eIF4E U2Os cells to vector controls using 3 different clones per condition. eIF4E-overexpressing cells used for sequencing had a ~3 fold increase in eIF4E relative to vector controls and thus within the range of elevation observed in cancer patients (>3–8 fold)^25,27,30–32^. We did not observe induction of splice factor mutations in SF3B1, U2AF1 or SRSF2 upon eIF4E overexpression^25^. eIF4E overexpression was not associated with global reprogramming of the transcriptome, with 402 differentially expressed genes (p_adj_<.05) in Vector versus 2FLAG-eIF4E U2OS cells of which 333 had > 2-fold change (GSE158728) ^33^. These transcripts were approximately equally distributed between elevated and repressed in eIF4E overexpressing cells (233 and 100 respectively) out of 5738 annotated transcripts observed with >10 TPM including both coding and non-coding RNAs ^21^ (GSE158728). Metascape analyses of these targets revealed GO terms such as; “p36 copy number variation syndrome,” “Defective B3GALT6 causes EDSP2 and SEMDJL1SEMDJL1” and “extracellular matrix organization” (Figure 1D & Supplemental Table 1). The top hits for protein-protein interaction enrichment analysis included “TNFR2 non-canonical NF-kB pathway”, “Defective B3GALT6 causes EDSP2 and SEMDJL1” and “positive regulation of DNA metabolic process” (Supplemental Table 2). The enrichment of NFκB signalling is interesting given eIF4E is a known target of NFκB^34^. Whether eIF4E’s impact on transcription is directly related to its physical association with active sites of transcription as described below remains to be determined. In all, eIF4E only influences steady state levels of ~6% of detected transcripts by >2-fold^25,27^.

Next, we assessed the impacts of NCBP2 overexpression on the transcriptome. We generated stably overexpressing 2FLAG-NCBP2 and vector U2OS cell lines monitoring 2 NCBP2 clones at 3 different passages and 3 different vector clones and conducted RNA-Seq to monitor changes to steady-state transcripts levels and splicing and compared these with the above eIF4E data. NCBP2 overexpression was 2.8 fold relative to vector (Figure 1B) comparable to the eIF4E-overexpressing cells GSE158728^27^. We note that CBC1A1 was removed from the analysis due to issues with library preparation of that sample which produced various mutations which were not present in a polyA purified sample from the same RNA (GSE300496 versus GSE295397 for polyA). For the remaining samples, we did not observe mutations in splice factors commonly associated with aberrant splicing: SF3B1, SRSF2, U2AF1 or U2AF2 using a 20% threshold for calling variants. Differential gene expression analysis revealed that 101 genes had altered steady state levels upon NCBP2 overexpression relative to vector cells (>2-fold, FDR <.05; 55 reduced and 46 increased in NCBP2 than vector) (Figure 1C and Supplemental Table 3) (GSE300496). This represents ~1.2% of the total 8331 annotated transcripts detected with >10 TPM. Metascape analyses revealed distinct enrichment patterns from eIF4E for NCBP2. Top GO terms for NCBP2 include “Signaling by Receptor Tyrosine Kinases”, “Malignant Mesothelioma”, and “Cell-cell adhesion” (Figure 1D and Supplemental Table 4) . The biological pathways driven by eIF4E and NCBP2 overexpression differed although they both had cancer phenotypes (Figure 1D). A comparison of transcripts altered at steady state by eIF4E and NCBP2 revealed 3 transcripts in common: *PALM3, INHBE,* and the long non-coding RNA *MSC-AS1* (Supplemental Table 5). Thus overall, eIF4E and NCBP2 drive distinct changes to steady state transcript levels.

To establish if NCBP2 and eIF4E produced redundant or divergent impacts on processing, we compared the splicing outcomes of NCBP2 and eIF4E overexpression. We assessed the impact of NCBP2 overexpression on splicing by analyzing the above RNA-Seq data with replicate Multivariate Analysis of Transcript Splicing (rMATS)^35^ (Supplemental Table 6). rMATS calculates the “inclusion level differences” for splicing events such as exon skipping (SE), inclusion of mutually exclusive exons (MXE), intron retention (IR) and alternative splice site usage (A3’SS and A5’SS). We note that the sign for eIF4E inclusion level difference was opposite^27^ to the 2FLAG-NCBP2 analysis reported here, which was modified for this report to match the directionality of the NCBP2 data. Here, a value of +1 for SE events indicated that the relevant exon is 100% included in 2FLAG-eIF4E or 2FLAG-NCBP2 cells and 0% in Vector cells. Using rMATS, a comparison of NCBP2 relative to vector revealed that 3894 splicing events for 2524 genes were altered (FDR-adjusted p-value <0.15; Inclusion differences of >0.05 or <−0.05) or using more stringent cutoffs (p-value <0.1, Inclusion level differences of >0.1 or <−0.1) we observed for 1825 events for 1402 genes (Figure 1E, Supplemental Table 6). The RNA-Seq experiments contained a total of 60564 annotated transcripts, 8331 of which had >10 TPM in all samples meaning that ~16–30% were differentially spliced in an NCBP2-dependent manner depending on the cutoffs used. Only three targets, *TIGAR, LGR5,* and *SL7A10,* were differentially expressed and underwent AS upon NCBP2 overexpression, suggesting that NCBP2-related alternative splicing (AS) is not generally correlated to mRNA levels under these conditions (Figure S1A). In terms of splicing targets, there are ~3.3 times as many genes than those modulated by eIF4E and ~4.4 times as many splicing events, as noted above: 886 splicing events of 762 genes in eIF4E-overexpressing U2Os (FDR-adjusted p-value <0.15; Inclusion differences of >0.05 or <−0.05) and 548 splicing events for 487 transcripts (p-value <0.1, Inclusion level differences of >0.1 or <−0.1) with 5738 annotated transcripts with >10 TPM^25^. Like NCBP2, only ~2% (13 transcripts) had both splicing and steady-state levels altered by eIF4E^27^. This gives a total of 8–13% of genes undergoing AS for eIF4E, and 16–30% observed for NCBP2. In all, the two cap-binding proteins elicited broad but not global impacts on splicing signatures and more restricted effects on transcript levels. Moreover, in both cases, their splicing targets are not enriched in targets which had altered steady state mRNA levels.

In terms of NCBP2, skipped exons (SE) were by far the most frequent event (~70%) followed by MXE events (~20%) with an approximately equal distribution between positive and negative inclusion level differences upon NCBP2 overexpression indicative of splicing reprogramming of selected RNAs rather than global alteration to splicing (Figure 1E). While there were some non-coding RNAs, coding RNAs predominated (Supplemental Table 6&7). As skipped exon events were the most common, we focused the remainder of our analyses on these events. Unsupervised hierarchical clustering analyses based on the inclusion levels across replicates revealed that SE events segregated into vector and NCBP2-overexpressing groups (Figure 1F). We noted no dependency of NCBP2-dependent splicing on exon length or GC content within introns (Figure S1B&C) but similar to our observations for eIF4E-overexpression, introns of alternatively spliced transcripts that were modulated by NCBP2 overexpression were longer by ~1.5 times or more than average introns (Supplemental Table 8). Consistent with previous studies in other cellular contexts^9,36^, we noted a bias towards the first three exons being targeted upon NCBP2-overexpression but note that other exons were also targeted (Figure 1G, left and Figure S1D, top). A reanalysis of eIF4E data employing the same strategy also demonstrated a bias for eIF4E transcripts for first, second and third exons for both SE and MXE (the second most frequent event); however, like NCBP2, other exons were also be impacted (Figure 1G, right and Figure S1D, bottom). Metascape analysis on NCBP2 AS targets revealed pathways related to cell growth such as “Cell Cycle”, “DNA metabolic process”, and “membrane trafficking” (Figure 1H; Supplemental Table 9). Consistently, NCBP2 overexpression also influenced alternative splicing of genes in similar pathways in cardiac cells related to cytoskeletal organization (astral microtubule organization, actin-filament based process, membrane organization) and the cell cycle (cell division, regulation of PLK1 activity at G2/M transition). In all, both NCBP2-dependent and eIF4E-dependent splicing is predominated by SE events with substantial amounts of MXE events in U2OS cells. Both cap-chaperone splicing programmes are characterized by a tendency to be in exons close to the 5’end and involve unusually long introns; but no correlation was observed regarding GC content in these introns or length of targeted exons (Figure 1& S1). Next, we established whether eIF4E and NCBP2 drove redundant splicing programmes as would be expected for cap-chaperone housekeeping activity or if cap-chaperones drove selective splicing programme. Strikingly, a comparison of their splicing signatures revealed only 365 transcripts in common or ~14% of the genes with altered splicing induced by eIF4E or NCBP2 (Figure 1I; Supplemental Table 10). An ontology analysis of genes with splicing altered by eIF4E, NCBP2, or both revealed strongly enriched pathways related to division e.g. “mitotic cell cycle” and “cell division”, in common with one another (Figure 1H; Supplemental Table 11). These support that while the targets may be different, there are common pathways driven by eIF4E and NCBP2. Interestingly, each group of alternate splice target genes were also involved in unique pathways; “DNA metabolic processes” in genes targeted by NCBP2, “response to ionizing radiation” in genes targeted by eIF4E, and “protein polyubiquitination” in genes affected by both proteins were highly enriched (Figure 1H).Despite the fact that the mRNA targets of eIF4E and NCBP2 share common properties, particularly a bias toward the 5’ end of exons, long introns and SE, and are involved in some of the same biological processes, these cap-binding proteins elicit splicing and transcriptomic signatures that diverge for >80% of affected transcripts supporting the SOCC model (Figure 1A, bottom). Consistently, the similarity in AS-dependent pathways between NCBP2 overexpression in U2OS cells and NCBP2 overexpression or knockdown in mouse cardiomyocytes^37^ (Figure S1E, 11–13% respectively) implies that the ability of NCBP2 to regulate AS of specific transcripts is conserved across species.

Our above assessment allowed us to identify ontological similarities and differences between eIF4E and NCBP2. Next, we established the number of overlapping splicing events to better quantify overlapping splicing mechanisms. We observed that only ~46 SE events (~2% of NCBP2 SE events) and 33 MXE events (~4%) (Figure 1I, Supplemental Table 12) were conserved between eIF4E and NCBP2 overexpressing cells (FDR < 0.15 Inclusion Level Difference < −0.05 or > 0.05). Thus, there were very few overlapping events between the two cap-chaperones consistent with the SOCCS model.

### Validation confirms cap-chaperone specific splicing events

**Figure 2:**
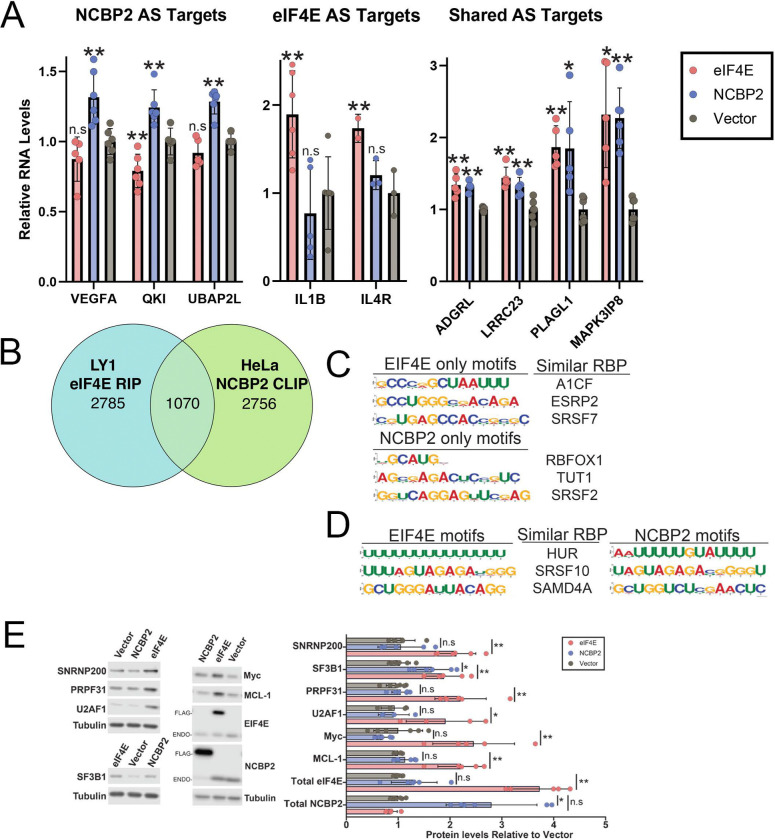
eIF4E and NCBP2 Interact with Distinct RBPs. **A)** Quantitative PCR showing alternative splicing following NCBP2 or eIF4E overexpression. **B)** Euler plot showing the overlap between eIF4E RIP targets from LY1 cells (blue) and NCBP2 iCLIP targets from HeLa cells (green). **C)** Significantly enriched motifs found in the introns of skipped exons from genes with alternative splicing only in eIF4E (top) or NCBP2 (bottom). RNA binding proteins recognition motifs similar to the enriched motifs are listed. **D)** RNA binding proteins with recognition motifs similar to motifs enriched in the introns of genes undergoing alternative splicing in eIF4E (left) or NCBP2 (right) overexpressing cells. **E)** Western blot showing the impact of eIF4E or NCBP2 overexpression on production of each other and selected splice. *Left* Representative blots probed as indicated. ENDO refers to endogenous NCBP2 or eIF4E and FLAG, the overexpressed form. *Right* Quantitation of 3–6 biological replicates for each condition are shown. For all panels, means, standard deviations and p-values relative to vector (Welch t-test) were calculated in PRISM. * = p-value < 0.05, ** = p-value < 0.005, n.s. = non-significant

Next, we validated splicing events selecting those specific to NCBP2, eIF4E or those in common using appropriate primers and RT-qPCR. For transcripts targeted by both eIF4E and NCBP2, we observed that *MAPK8IP3*, *LRRC23, PLAGL1, ADDGRL*, were common targets as predicted by rMATS. It should be noted that MAPK8IP3 does not appear on the current list of alternately spliced targets of NCBP2, while it did in a previous analysis conducted from sequencing libraries of the same samples prepared using a poly-A pulldown; thus highlighting the limitations of RNA-sequencing in capturing the entire landscape of RNA modifications. For the eIF4E-only group, *IL1B* and *IL4R* previously validated eIF4E splicing targets^27^, were modulated by eIF4E but not NCBP2 (Figure 2A). For the NCBP-dependent group, *VEGFA*, *QKI*, and *UBAP2L* was targeted by NCBP2 but not eIF4E consistent with the rMATS data (Figure 2A).

### eIF4E and NCBP2 physically interact with a subset of AS target transcripts and pre-mRNAs

While eIF4E and NCBP2 are cap-binding proteins, they did not bind to all capped-mRNAs in the nucleus: e.g eIF4E immunoprecipitated with ~3800 RNAs^15^ and NCBP2 with ~2500 mRNAs in cardiomyoctyes and ~3800 in HeLa cells (Figure 2B)^36,37,38^. A comparison of eIF4E and NCBP2 RIP-Seq datasets revealed that 1070 transcripts or ~30% overlapped between these two groups, indicating that most of these RNAs only physically associated with one of these chaperones. This is less than the 45–60% overlap we see between the full LY1 and HeLa transcriptome sequenced in the respective studies, suggesting the differences between the two cap binding protein targets stem from selective targeting of RNAs and not just cell line differences. At the splicing event level, only 79 events from 74 genes were in common between eIF4E and NCBP2 and of these 17 were found in the LY1 RIP and 29 were found in the HeLa CLIP. Moreover, ~36% (898 transcripts) of AS targets of NCBP2 also physically interacted with NCBP2 in HeLa cells^36^ (Figure S2A) similar to the ~34% eIF4E-dependent AS targets were found in the eIF4E RIPs^27^. Indeed, in none of the cases were all RNAs in the RIPs for the respective cap-chaperone AS targets and in some cases AS targets were not found in RIPs indicative of both direct and indirect effects of NCBP2, as observed previously for eIF4E^27^.

### Conserved RNA elements found in targeted transcripts provide insight into cap-chaperone selectivity

To elucidate the molecular basis for the selectivity of these cap-chaperone proteins, we investigated whether AS targets contained sequences that could act as USER codes for the respective splicing programmes. We utilized the MEME suite program XSTREME to identify enriched motifs in the sequence of SE events and surrounding introns in three groups: eIF4E-dependent only, NCBP2-dependent only, or shared. We noted that analysis of the 46 shared skipped exon events did not reveal any enriched motifs in the exon or 5’ intron sequences, likely due to the low number in this group. However, we did identify the recognition motif of SRSF10 in the 3’ introns. In all three groups, we did not find enriched motifs in the exonic sequences, but we observed motifs similar to known RBP recognition sequences as well as motifs without known function in the upstream and downstream introns (Figure 2C–D; Supplemental Table 13 and 14).

XTREME analysis of AS targets revealed enriched sequence motifs present in introns specific to the eIF4E-dependent and NCBP2-dependent groups (Figure 2C and Figure S2B, right). For example, the eIF4E-dependent group was enriched for the binding sites similar to consensus binding sites for A1CF, ESPR2, MBNL1 and SRSF7. A1CF is a component of the apolipoprotein B mRNA editing complex, ESRP2 is involved in splicing *CD44*, MBNL1 acts in specific splicing events including *TNNT2* through competition with U2AF2, and SRSF7 acts in specific splicing events including splicing of *MAPT/Tau*^39–41^. Interestingly, eIF4E physically interacted with *CD44* transcripts, modulated their splicing, and promoted their mRNA export and translation^27,42^. Also, HuR/ELAVL1 was identified in the shared groups. Consistent with these findings, HuR binds eIF4E in an RNA-dependent manner and several eIF4E AS targets are known to be enriched in ARE elements^27^. Unique to the NCBP2-dependent group were binding motifs similar to the following splicing and general RNA processing factors: SART3, SFPQ, FXR2, RBFOX1, RBM5, TUT1, SRSF2, and BRUNOL5/CELF. Gene ontology analysis revealed that many of the RBPs with recognition motifs similar to the enriched motifs from both groups are involved in mRNA splicing and other RNA metabolic processes (Figure S2B, left). Further evidence that eIF4E and NCBP2 distinct effects on splicing may stem from unique responses from RBPs comes from the degree of enrichment of RBPs amongst the AS targets. RNA binding proteins make up 1712 of the NCBP2 AS targets, representing much stronger enrichment than the 442 RBPs in eIF4E AS targets (Figure S2C). In both cases this is the majority of genes that are undergoing alternative splicing, representing ~67% of NCBP2 and ~58% of eIF4E targets. RNA binding proteins that play a role in RNA metabolism changing as a result of alternative splicing also provides another possible explanation for the number of genes that are affected by NCBP2 or eIF4E but do not physically interact with them (Figure S1F and S2A).

Many of the splicing factors whose motifs appear in these introns for all three groups play a role in 5’ splice site selection, including SRSF1 and SRSF10. Members of the heterogenous nuclear ribonucleoprotein (hnRNP) family HNRNPC, HNRNPLL, and HNRNPH2 which also act in splicing appear in both the NCBP2 and eIF4E introns, while HNRNPK appears in the NCBP2-dependent and shared gene introns and HNRNPA2B1 appears only in NCBP2-dependent introns. The similarity in splice factor motifs is echoed in the similar enrichment of splice factors amongst the alternative splicing targets of eIF4E and NCBP2 (Figure S3C). Some motifs similar to general RNA-binding sequences were conserved in the introns of all three groups: SAMD4A, a translational repressor, and PABPC3 and PABPC4, factors that bind the poly-A tail of mRNA. To determine if any of these motifs were found in distinct positions within the introns, we utilized FIMO from the MEME suite toto determine the location of the enriched motifs from XSTREME in the upstream and downstream introns of each group. We then calculated the relative position of the motifs with q-value < 0.05 within the intron. In aggregate, none of the motifs examined demonstrated a position preference within the introns (Figure S2D, using the SRSF10 motifs as an example). Analysis of targeted exons revealed less enriched RBP motifs than introns. There were no significant enriched motifs identified in the exonic sequences for the eIF4E-dependent and shared groups. However, there were two significant motifs identified in the sequences of the skipped exons shared in the NCBP2-dependent groups. One of these motifs is similar to the recognition motifs of the RNA binding protein SRSF1, while the other is not like any known RBPs (Figure S2E). In all, our analyses indicate that intronic sequences predominate and thus are more likely to influence cap-chaperone specification.

### NCBP2 and eIF4E differentially influence production of the splicing machinery

Previously it was shown that eIF4E manifested splicing changes through two simultaneous effects: its physical association with some splicing substrate mRNAs and the splicing machinery, as well as enhanced production of the some components of the splicing machinery including SF3B1, U2AF1, SNRNP200 and PRPF31^27^. Here, we examined if NCBP2 similarly impacted splice factor production. For this comparison it is important to note that eIF4E and NCBP2 overexpression relative to respective vector controls were both ~3-fold and thus comparable (Figure 2F). Notably, this level of overexpression is in the mid-range of expression seen in eIF4E and NCBP2 overexpression cancers and is thus physiologically relevant^30,43^. Neither cap-chaperone modulated production of the other indicating that there was no feedback on the levels of cap-chaperones to limit their overall abundance (Figure 2E). eIF4E, increased protein production of its mRNA export and translation targets: MYC by ~2.5-fold and MCL1 by ~2.2-fold relative to vector while NCBP did not alter production of these. Interestingly, NCBP2 did not increase production of U2AF1, SNRNP200 or PRP31 relative to vector controls while eIF4E elevated these by ~2-fold consistent with previous work^27^ (Figure 2E). NCBP2 elicited an increase in SF3B1 protein levels by ~1.7-fold relative to vector while eIF4E has ~2-fold change consistent with previous studies^27^. Also, neither eIF4E nor NCBP2-overexpression substantially altered levels of UsnRNAs by RT-qPCR consistent with previous studies^27^ (Figure S2E). Thus, eIF4E and NCBP2 overexpression elicited distinct impacts on the production of spliceosome components (SF3B1, PRPF31 and SNRNP200) as well as on factors that indirectly influence splicing e.g. MYC^44^. In all, these two cap-chaperones produced different splicing landscapes which likely contributed to their distinct splicing signatures.

### eIF4E physically associates with sites of active transcription

**Figure 3.**
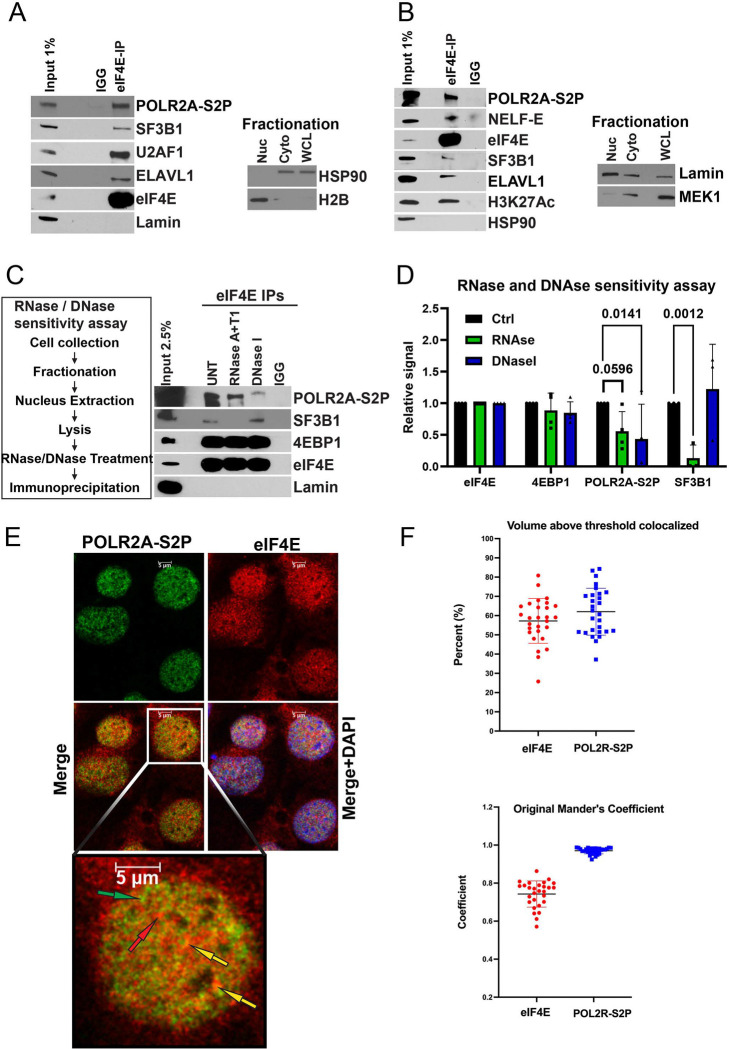
A fraction of eIF4E interacts with active sites of transcription. **A)** Endogenous eIF4E co-immunoprecipitated with POLR2A-S2P in the nuclear fraction of U2OS cells. Immunoprecipitations (IP) were carried out using nuclear lysates and anti-eIF4E antibody (eIF4E-IP) or appropriate IgG control (IGG), carried out at least three independent times with one representative experiment shown. Samples were analyzed by western blot (WB) using antibodies as indicated. Fractionation controls for HSP90 for the cytoplasmic fraction (Cyto), histone 2B (H2B) for the nuclear fraction (Nuc) are shown. Whole Cell lysis (WCL). **B)** Endogenous eIF4E co-immunoprecipitated with POLR2A-S2P in crosslinked nuclear fractions using NEXSON, one representative experiment is shown of three biological replicates. **C)** The impact of DNAse and RNAse on the eIF4E-POLR2A-S2P interaction were analyzed by western blot. **D)** Quantification of three or more biological replicates from C) showing the relative signal of each protein in control (black), RNase-treated (green), and DNase-treated conditions (blue) (p-values are provided using Student t-test in PRISM, all comparisons non indicated were non-significant). **E)** Confocal micrographs of U2OS cells stained with antibodies against eIF4E (red) and POLR2A-S2P (green), with nuclei counterstained with DAPI (blue) and overlay in yellow (merge). Single section through the plane of the cell is shown. Arrows indicate examples of eIF4E-only (red), POLR2A-S2P-only (green), or co-localized (yellow) signals **H)** Quantitation of colocalization using Imaris, data were collected as Z-stacks for >25 cells. Percentage colocalization over the cell volume and the Manders coefficient were calculated in Imaris and processed in PRISM. Each data point represents a single nuclei.

Next, we examined whether other mechanisms also contribute to the cap-chaperone-dependent splicing profiles. NCBP2 is well known to physically associate with active transcription machinery^6^. Thus, another possible basis for the different transcriptome and splicing signatures between cap-chaperones is that NCBP2 associates with the transcriptional machinery while eIF4E played roles in post-transcriptional RNA processing. To investigate this possibility, we examined whether endogenous eIF4E associated with transcription sites using antibodies to RNA polymerase II phosphorylated at Serine 2 (POLR2A-S2P) to mark active transcription^45,46^. eIF4E immunoprecipitations from nuclear lysates revealed interactions between endogenous eIF4E and POL2RA-S2P (Figure 3A). eIF4E interacted with positive controls HuR/ELAV1, NELF-E, splicing factors SF3B1 and U2AF1 but not the negative control Lamin nor were any of these proteins found in the IgG controls. H2B and Lamin served as fractionation controls for the nucleus and MEK1 and HSP90 for the cytoplasm (Figure3A&B).

To ensure that the eIF4E-POL2RA-S2P interaction did not arise due to reassortment during immunoprecipitation, we employed the nuclear extraction by sonication method (NEXSON)^47^ which involves crosslinking cells prior to isolation of nuclei by sonication and additionally better solubilizes the transcription machinery (Figure 3B). Like native (not crosslinked) nuclear lysates, we observed that endogenous eIF4E bound to a fraction of POLR2A-S2P but not to the negative control . We also observed that eIF4E bound to another marker of active transcription, acetylated histone H3K27 (H3K27-Ac)^48^ and NELF-E^49,50^, a component of the RNA polymerase complex responsible for pausing which is important for coordinating transcription with splicing^5,6,50^ (Figure 3B).

To assess whether eIF4E required intact chromatin and transcripts for this physical interaction, we monitored DNAse and RNAse sensitivity of the eIF4E-POL2RA-S2P interaction in uncrosslinked nuclear fractions. Nuclear lysates were treated with DNAse I or RNAses T and A1 followed by eIF4E immunoprecipitation. Representative blots and quantitation are shown (Figure 3C&D). DNAse I treatment reduced the eIF4E-POL2RA-S2P interaction by ~11-fold relative to untreated controls and RNAse treatment by ~1.5 fold. eIF4E directly interacted with 4EBP1 and this interaction was not disrupted by either DNAse or RNAse, as expected. These factors were not observed in the IgG controls and Lamin served as a negative control. Immunoprecipitation efficiency between experiments was controlled by normalizing to the efficiency of eIF4E immunoprecipitation of itself in the same nuclear lysate which we note were not impacted significantly by either DNAse or RNAse (Figure 3D). Finally, the interaction of eIF4E with SF3B1 was RNAse-dependent as shown previously^27^ and interestingly, not reliant on chromatin as seen by insensitivity of the eIF4E-SF3B1 interaction to DNAse I treatment (Figure 3C–D). In all, the physical interactions between eIF4E and POL2RA-S2P depended on the presence of DNA and RNA consistent with a reliance on intact transcription complexes and proximity to RNA processing events.

To ensure the interaction was observed in intact cells, we employed immunofluorescence and confocal laser microscopy collecting Z-stacks throughout the cell volume. We observed that eIF4E is localized in spheroid nuclear structures as well as diffusely throughout the nucleoplasm and cytoplasm consistent with previous studies ^13,22,24,51,52^(Figure 3E). POL2RA-S2P was similarly found in puncta as well as a diffusely throughout the nucleus and excluded from the cytoplasm. Quantification of colocalization within the nucleus was performed using Imaris. We observed that 57+/−11% of eIF4E co-localized POL2R-S2P and conversely 62+/−12% of POL2RA-S2P colocalized with eIF4E (Figure 3F). The average original Mander’s overlap coefficient which provides the correlation of absolute intensity was 0.74 for eIF4E and >0.9 for POL2RA-S2P in eIF4E/POL2RA-S2P co-staining indicating this was non-random. Note that for quantitation, secondary antibody staining served as the background signal for subtraction. In all, we observed that a fraction of endogenous eIF4E physically associated with transcriptionally active RNA polymerase II and other markers of active transcription.

### Physical separation of eIF4E and NCBP2 is a mechanism for cap-chaperone specification

**Figure 4.**
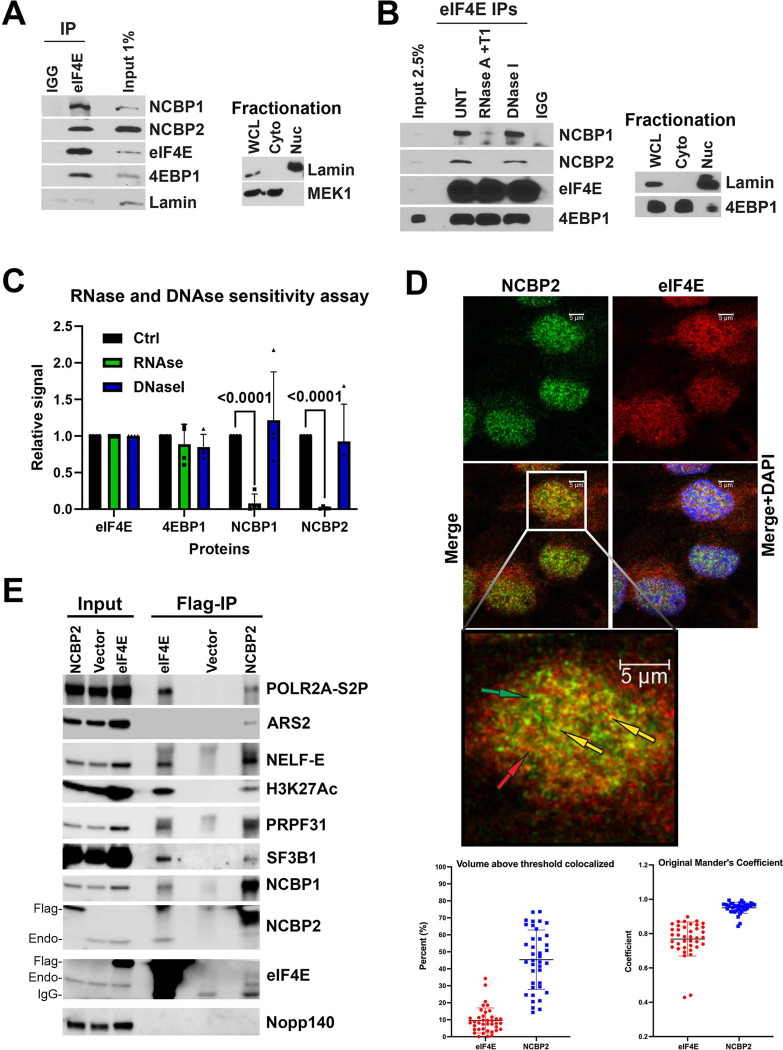
Mechanisms for eIF4E and NCBP2 dependent splicing. **A)** Endogenous eIF4E co-immunoprecipitated with NCBP1 and NCBP2 in nuclear fractions from U2OS cells. Immunoprecipitations (IP) were carried out using U2OS nuclear lysates and anti-eIF4E antibody (eIF4E-IP) or appropriate IgG control (IGG), carried out at least three independent times, with a representative experiment shown. Fractionation controls included Lamin (nuclear fraction, Nuc), MEK1 served as controls for the cytoplasmic fractions (Cyto). Whole Cell lysis (WCL). **B**) The impact of the RNAse and DNAse treatment on of the interaction between endogenous eIF4E and NCBP2 and NCBP1. IPs were performed using nuclear lysates from U2OS cells with or without RNase A/T1 or DNase I treatment prior to IP (as in Figure 3C). IP samples, along with input, were analyzed by western blot using the indicated antibodies. **C)** Quantification of three biological replicate experiments showing the relative signal of each protein in control (black), RNase-treated (green), and DNase-treated conditions (blue) (p-values are provided using Student t-test in PRISM, all comparisons non indicated were non-significant). **D)** *Top* Confocal micrographs of a single section through the plane of U2OS cells stained with antibodies against endogenous eIF4E (red) and NCBP2 (green), overlay (yellow) and nuclei with DAPI (blue). Arrows indicate examples of eIF4E-only (red), NCBP2-only (green), or co-localized (yellow) signals. *Bottom* Quantitation of colocalization using Imaris, data were collected as Z-stacks. Percentage colocalization over the cell volume and the Manders coefficient are shown. Each point represents a single nuclei. Data were collected as Z-stacks for >25 cells and processed in PRISM. **E)** FLAG Ips for nuclear lysates of NCBP2- or eIF4E-overexpressing cells showing overlapping but distinct complexes were formed. Representative experiments of two different eIF4E and NCBP2 clones in biological replicates are shown. Vector control cells and probing for NOPP140 served as negative controls.

Given that a fraction of both eIF4E and NCBP2 were found at active sites of transcription, we explored whether differential designation of mRNA fates arose due to partial, or even complete, separation of eIF4E and NCBP2 in the nucleus at different transcription sites. To assess spatial association, we monitored localization of eIF4E and NCBP2 by confocal microscopy (Figure 4D). We observed that 46+/−18% of NCBP2 with eIF4E and 10+/−8% of eIF4E with NCBP2. Thus, eIF4E and NCBP2 existed mostly separately but could be found together. Indeed, endogenous eIF4E immunoprecipitated with NCBP2 and its co-factor NCBP1 in native nuclear fractions from U2OS cells (Figure 4A). No immunoprecipitation was observed for Lamin and no interactions were observed in the IgG controls. NCBP1 and NCBP2 interactions with eIF4E were reduced by 27-fold and 10-fold upon treatment with RNAse, respectively (Figure 4B&C). By contrast, DNase I treatment did not significantly reduce the binding of eIF4E to either NCBP1 or NCBP2. The interaction between eIF4E and 4EBP1 was insensitive to either DNAse or RNAse as expected given its direct interaction with eIF4E^27^ providing a negative control. Thus, the eIF4E-NCBP2/NCBP1 interaction was RNA-dependent suggestive that these cap-chaperones localized to sites of mRNA processing such as transcription factories which are disrupted by RNAse treatment but did not require DNA. In all, the mainly distinct localization of eIF4E and NCBP2 likely contribute to their distinct modulation of transcriptomic and splicing signatures.

### eIF4E and NCBP2 bind common transcriptional machinery but only NCBP2 binds the ARS2 adaptor

Several key factors are known to underpin the capacity of the NCBP2/NCBP1 complex to associate with the processing machinery including ARS2, also known as SRRT^6^. ARS2 mediates interactions between NCBP2/NCBP1-capped RNA complexes early in transcription. ARS2 plays role in recruiting many factors including the splicing and mRNA export machinery to the NCBP2/1 complexes bound to mRNAs^6,53^. We examined the possibility that ARS2 was specifically interacting with NCBP2 and not eIF4E complexes as another possible mechanism underlying their distinct impacts on splicing. Interactions were examined in two different eIF4E and NCBP2 stable cells lines each in biological duplicate and crosslinked lysates prior to IP to avoid reassortment (Figure 4E). We observed that FLAG-eIF4E interacted with NCBP1 and NCBP2 as expected as well as NELF-E and H3K27Ac as seen with the endogenous splicing machinery (Figures 3&4). As expected, FLAG-NCBP2 was complexed to ARS2, NELF-E, eIF4E and POL2RA-S2P^6^. Interestingly, FLAG-eIF4E did not interact with ARS2. Thus, the ARS2 containing mechanism utilized by NCBP2 to interact with the splicing machinery is not shared between the cap-chaperones. In this way, these factors have distinct partner protein configurations underlying the different splicing landscapes.

## Discussion

Direction of nuclear mRNA fate through the m^7^G cap was considered the sole purview of NCBP2 (Figure 1A). This model was based upon the assumption that NCBP2 was the only nuclear cap-binding protein and that its activity was constitutive i.e. functioning automatically (Figure 1A). However, eIF4E and NCBP2 co-exist in the nuclei of plant, yeast and animal cells^9,10,13,14,22,29^. We showed that each cap-chaperone drove alternative splicing for thousands of mRNAs with only 365 shared transcripts with 79 common events. Thus, the predominant purpose for coexistence of cap-chaperones in the nucleus is not functional redundancy but rather, designation of specific mRNAs for processing thereby embodying a novel regulatory control point in gene expression as outlined by the SOCCS model (Figure 1A, bottom panel). In this model, cap-chaperones designate specific mRNAs for regulation at processing steps such as splicing modulating their fate. This is the first time that cap-chaperone selection of transcripts has been implicated as regulatory point in gene expression. It is also striking that a relatively small percentage of transcripts undergo AS for either cap-chaperone eIF4E (8–13% of transcripts) or NCBP2 (16–30% of transcripts) . This is suggestive that more cap-chaperones exist.

**Figure 5:**
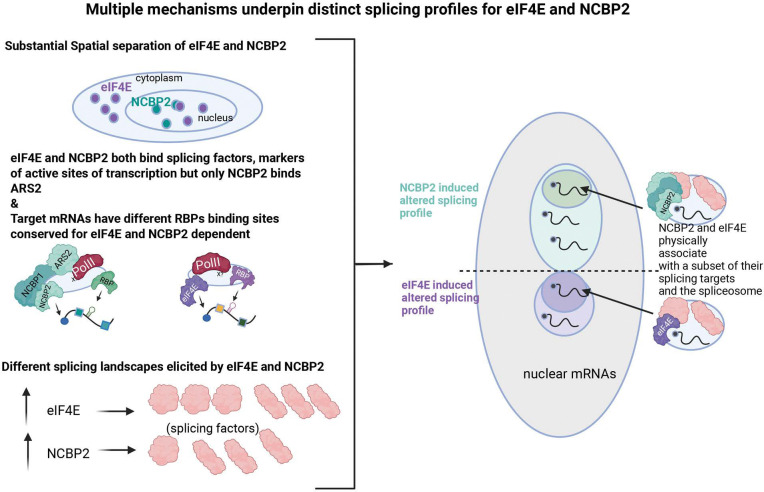
Multiple mechanisms underpin distinct splicing profiles for eIF4E and NCBP2. *Left* eIF4E and NCBP2 have some nuclear co-localization, but are largely separate from one another. However, they can both binding splicing factors and markers of active transcription with notable differences such as ARS2 only binding NCBP2. Together with the unique effects on the splicing factor landscapes, this leads to changes in the splicing profile of distinct sets of mRNAs, with small overlap.

We identified several molecular mechanisms underpinning dependency on cap-chaperones and subsequent generation of distinct splicing profiles (Figure 5). Indeed, our studies indicate that there are multiple, contemporaneous mechanisms at play leading eIF4E and NCBP2 to influence splicing of distinct subsets of pre-mRNA splice substrates. We identified specific motifs within transcripts that could lead to dependency on a specific cap-chaperone. For instance, RBP-binding motifs such as ESPR2 and A1CF were found only in the eIF4E-dependent splicing groups while motifs similar to binding sites for SRSF2 were only found in introns in the NCBP2-senstive group (Supplemental Table 13; Figure 2C&D). This supports a model whereby eIF4E and NCBP2 interact (directly or indirectly) with RBPs to support cap-chaperone specific complexes. We also found that there were differences in complexes formed with each chaperone in the nucleus, where ARS2 only associates with NCBP2 (Figure 4E). Further, we explored the possibility that spatial separation of eIF4E and NCBP2 contributed to different profiles. While eIF4E, like NCBP2, interacted with active transcription sites, we observed substantial physical separation of eIF4E and NCBP2 indicative of these acting at distinct transcription sites (Figure 4D). This physical separation provides another molecular basis for the different splicing profiles elicited by these cap-chaperones.

Next, we investigated the indirect effects of eIF4E and NCBP2 on the splice factor landscape which could contemporaneously influence splicing even for mRNAs not physically associated with the given cap-chaperone. We examined components of different UsnRNPs to establish if any effects were restricted to one complex or by contrast influenced several splicing steps. Consistent with previous studies, eIF4E increased the production of the following factors 2–3 fold: SF3B1 (U2 snRNP), U2AF1 (U2 snRNP), PRPF31 (U4/U6 snRNP) and SNRNP200 (U5 snRNP) ^27,51,54^ (Figure 2E). By contrast, NCBP2 did not modify production of U2AF1, SNRNP200, or PRPF31, but did elevate SF3B1 by nearly 2-fold similar to eIF4E (Figure 2E). Further, transcripts coding for splicing factors and RNA binding proteins were also found among alternatively spliced genes and were more strongly enriched in NCBP2 overexpression, where they made up ~67% of splicing targets. This suggests that eIF4E and NCBP2 by modulating splice factor production change the splicing landscape in unique ways to further support impacts on specific transcripts. Finally, it was also possible that there is a cellular mechanism to limit cap-chaperone levels where overexpression of one cap-chaperone reduced endogenous levels of the other. However, we noted that neither cap-chaperone influenced the other’s steady-state protein levels (Figure 2F). Thus, we identified multiple mechanisms which in aggregate contribute to differential specification of mRNAs to a given cap-chaperone.

The traditional view predicts that modulation of NCBP2 would influence splicing globally (Figure 1A); however, we observed only 16–30% of the detected transcriptome was modulated by NCBP2. Consistent with these results, recent reports demonstrated that NCBP2 overexpression influenced the splicing of ~1000 transcripts in mouse cardiac cells^37^, 544 of which were in common with those found here (Figure S1E). Similar to our findings, this study showed that NCBP2 principally impacted SE events which only rarely corresponded to altered transcript levels^37^. The splicing effects of NCBP2 in cardiac cells were sufficient to retard heart maturation *in vivo* demonstrating the biological relevance of this pathway^37^. While eIF4E elevation is well known to contribute to multiple cancer phenotypes^19,55,56^, recent studies have also implicated increased NCBP2 levels as a contributor to malignancy^43,57,58^ supporting the biomedical relevance of this regulatory point. The recent discovery of NCBP3 as another nuclear cap-binding protein^5^ is easily incorporated into the SOCCs model and suggests that understanding the chaperoning of capped-RNAs through the gene expression steps may be a multi-body rather than only a two-body problem. Indeed, it is theoretically possible that mRNAs may even be exchanged between cap-chaperones at different stages of processing constituent with our observation that 365 transcripts are shared between eIF4E and NCBP2.

In all, we discovered an unforeseen step in gene expression which stands in stark opposition to the current paradigm (Figure 1A, Figure 5). Specification of cap-chaperones for a given transcript as in the SOCCs model constitutes a previously unknown point for biological control. Incorporating this model into our current understanding of gene expression will better enable us to target cap-chaperone proteins and predict the impacts of targeting these in patients both in terms of potential compensatory mechanisms and biological impacts. In all, our findings support directed cap-chaperoning of transcripts which incorporates a cap-chaperone specification step to determine mRNA fate.

## Materials and Methods

### Cell culture

U2OS cells were obtained from ATCC and maintained at 37°C and 5% CO2 in Dulbecco’s modified Eagle’s medium (DMEM) (ThermoFisher Scientific) supplemented with 10% fetal bovine serum (FBS) (ThermoFisher Scientific) and 1% penicillin–streptomycin (ThermoFisher Scientific). pcDNA-2FLAG-eIF4E and Vector stable cell lines were generated previously^15,16,21^, and maintained as U2OS cells with the addition of G-418 (1 mg/ml, Wisent Bioproducts). NCBP2-pRETS was a kind gift of A Segref and I Mattaj. pcDNA-2FLAG-NCBP2 was generated and included the entire coding region of NCBP2 confirmed by DNA sequencing. Stable FLAG-NCBP2 cell lines were generated as described^15,16,21^. Cell lines were authenticated using STR profiling (Wyndham Forensic Group). Cultured cells were routinely checked to ensure that there was no mycoplasma contamination by PCR^59^.

### Western blot analysis.

Western blot analysis was performed as described previously^42^. Blots were blocked in 5% milk in TBS–Tween 20. Primary antibodies were diluted in 5% milk. Quantification of blots was carried out using FIJI and plotted in PRISM. Antibodies used are found in Supplemental Table 15.

### Cellular fractionation

About 5 × 10^7^ U2OS cells were collected and washed twice in ice-cold PBS (300 × g for 3–5 min) and then resuspended with slow pipetting in 0.5 ml of lysis buffer B (10 mM Tris (pH 8.4), 140 mM NaCl, 1.5 mM MgCl2, 0.25% Nonidet P-40, 1 mM DTT, 100 U/ml RNase inhibitors). The lysate was centrifuged at 1,000 × g for 3 min at 4°C, and the supernatant (cytoplasmic fraction) was transferred into a fresh microtube. The pellet (nuclear fraction) was resuspended in 1 Volume of lysis buffer B and transferred to a round-bottomed polypropylene tube, and 1/10 volume of detergent stock (3.3% sodium deoxycholate, 6.6% Tween 40 in DEPC H2O) was added with slow vortexing (to prevent the nuclei from clumping) and incubated on ice for 5 min. The suspension was transferred to a microtube and centrifuged at 1,000 × g for 3 min at 4°C. Supernatant (post-nuclear fraction) was added to the cytoplasmic fraction. Clonal stable FLAG-eIF4E overexpressing cell lines were generated previously and NCBP2 was cloned into the same FLAG vector which was confirmed by sequencing to be oriented correctly and have no mutations and clonal FLAG-NCBP1 stable cell lines generated as described for eIF4E^27^.

### RNA extraction and quantitative PCR.

DNAse treated RNA samples (Direct-zol RNA Miniprep Kit, Zymo Research) were reversed transcribed using MMLV reverse transcription (ThermoFisher Scientific), qPCR analyses were performed using SensiFastSybr Lo-Rox Mix (Bioline) in Applied Biosystems QuantStudio thermal cycler using the relative standard curve method (Applied Biosystems User Bulletin #2). All conditions were previously described^27^. Primers used are available in Supplemental Table 16.

### RNA Sequencing and Differential Gene expression analysis for total RNAs

RNAs isolated nuclear extracts from five FLAG-NCBP2 and three FLAG vector stable cell lines underwent ribodepletion using the KAPA RNA HyperPrep Ribodepletion Kits (Roche) according to the manufacturer’s instructions. All libraries were subjected to flow cell high-output (75 cycles) singe-end sequencing on an Illumina NextSeq 2000 Sequencer at the genomics facility at the Institute for Research in Immunology and Cancer (IRIC) at the University of Montreal. RNA-sequencing reads from cells expressing 2FLAG-NCBP2 or vector were trimmed using Trimmomatic version 0.35^60^ and aligned to the human genome GRCh38 using STAR version 2.7.1a^61^ with gene annotations from Gencode version 32 to match previous data for eIF4E. Data are deposited as GSE300496. Differential expression analysis was conducted using DESeq2 version 1.30.1^62^. Genes were considered to be significantly differentially expressed if they were changing more than 2-fold (log_2_FoldChange > 1) and had a FDR-adjusted p-value (p_adj_) < 0.01 unless otherwise noted.

### Alternative Splicing Analysis

Alternate splicing analysis was conducted on the STAR-aligned 2FLAG-NCBP2 overexpression and vector U2OS cells using rMATS version 4.3.0^63^ using the following settings: -t paired – nthread 20 –readLength 101 –variable-read-length –libType fr-unstranded. For the following analyses using both the NCBP2 U2OS rMATS results and the previously published eIF4E U2OS rMATS analysis^27^, alternate splicing events were considered significant if FDR < 0.15 and the absolute inclusion level difference > 0.05. The inclusion level difference of eIF4E rMATS was multiplied by −1 to make it consistent with the NCBP2 rMATS, where a positive inclusion level difference indicates higher inclusion in the overexpression samples. Coordinates of the skipped exons undergoing significant changes in each group were extracted from the rMATS results. Introns surrounding the skipped exons were defined by the sequence between the skipped exon and the upstream exon end coordinate or downstream exon start coordinate as recorded in the rMATS output. Shared skipped exon events were matched by identifying events with identical gene names, exon start sites and exon ends while shared mutually exclusive exon events were identified based on gene name and start coordinates of the two mutually exclusive exons.

### Enrichment analysis

A list of human RNA binding proteins was retrieved from RBP2GO^64^ and a list of splice factors was retrieved from the spliceosome database^65^, both in July 2025. The enrichment of proteins from these lists in the alternatively spliced genes was calculated using the enrichment function of the *bc3net* R package. Genes with a TPM > 10 in the respective overexpression was used as the reference gene list, with the shared AS targets using the NCBP2 reference gene list.

### Motif analysis

Sequences for the skipped exons and surrounding introns were retrieved via the BSgenome R package (version 1.74.0) from the BSgenome.Hsapiens.UCSC.hg38 version 1.4.5 genomic sequence collection (https://bioconductor.org/packages/BSgenome). Intron sequences contained 10nt of the skipped exon on the respective end of the intron to identify any motifs that span the intron-exon boundary. Sequences were provided to webserver of the MEME suite for motif identification via XSTREME (version 5.5.7)^66^ using default settings with the sequence interpreted as RNA and the human RNA binding protein motif compendium^67^ used to determine similar motifs. To determine the position of the motifs we provided the top 5 motifs for each group, and the sequences of the introns from that group, to FIMO version 5.0.5^68^ using default options. The FIMO results were filtered to q-value < 0.05 and the relative start position within the intron for each identified motif was calculated. Logos of the identified motifs were generated via Ceqlogo version 5.0.5 of the MEME suite^69^.^69^. Gene ontology analysis of RNA binding proteins identified during motif analysis was conducted using the biological process PAN-GO human gene functionome^70^.

### Position analyses of events in transcripts.

Previously, we normalized location by the total number of exons so that the x-axis represented the location in percent of the total gene for an exon. Here, we report the x-axis by the actual exon number. For SE events, the location of the skipped exon is reported. For MXE events, the number of the 5’-most exon that is involved in the mutual exclusion and for RI events, the location of the exons just 5’ of the retained intron.

### Immunoprecipitation with and without RNAse and DNAse treatment

Cells were collected and fractionated as described previously^71^. After fractionation, nuclei have been lysed by sonication (20% power, 3×6s with Sonic Dismembrator Model 500, Fisher, Max Output 400W) in 0.5 ml per 4×10^7^ cells of NT-2 buffer supplemented with 1× protease inhibitors. Lysates have been cleared by centrifugation at 10,000 × g 10 min at 4°C and transferred into a clean tube, and the concentration determined by BCA assay before being adjusted to 1 mg/ml. For RNAse treatment, 1mg of nuclear lysate has been incubated at RT for 20 min with RNase A (EN0531) at 20μg/ml final and RNase T1 at 1U/μl (EN0542).

For DNaseI treatment, 1/10^th^ of DNase I buffer and 10U/ml of DNase I (invitrogen: 18047019) was added to 1 mg of extract and incubated at room temperature for 20min. Untreated or treated lysates were cleared with 30 μl of Dynabeads G per mg of extract at 4°C for 40 min and centrifuge at 10,000 × g 10 min at 4°C. For 1 mg IP, 33 μl of Dynabeads G (Invitrogen) were preincubated with 10 μg of anti-eIF4E (rabbit, MBL) or rabbit IgGs at RT for 20 min. After five washes, beads are resuspended with precleared lysate and incubated with rotation at 4°C o/n. IPs were washed one time with NT-2 buffer supplemented with 1 mg/ml Heparin and five times with 1 ml of NT-2 buffer and resuspended in 2× LB and incubated at 95°C for 10 min. Samples have been resolved by SDS–PAGE and visualized by Western Blot. Quantification of immunoprecipitation was carried out using Image Lab 6.1.0 build 7 (Bio-Rad) and plotted in PRISM 10.4.2. Differences in co-immunoprecipitation (Co-IP) signal between conditions were analyzed using two-way analysis of variance (ANOVA), with treatments (Ctrl, RNase, DNAse) and protein partner of eIF4E as independent variables. Post hoc comparisons were conducted using Dunnett’s multiple comparisons test when appropriate. Data were presented as mean ± SEM only significant differences.

### NEXSON Immunoprecipitation

Nuclei Extraction by sonication (NEXSON) has been firstly described in Arrigoni et al.^47^. Briefly, U2OS cells are resuspended by trypsinization, collected at 1200 rpm for 5 min at 4°C, and crosslinked in 1% formaldehyde (10 min, RT). Crosslinking is quenched with 125 mM glycine (5 min, RT), and cells are centrifuged at 1000g for 5 min at 4°C and washed twice with PBS. For nuclei extraction, cells are resuspended in Farnham Lab Buffer(5 mM PIPES pH 8.0, 85 mM KCl, 0.5% IGEPAL CA-630, supplemented with proteases inhibitors), sonicated with Bioruptor^®^ Plus, 3 cycles of 15s on/30s off, 4°C, and collected by centrifugation at 1000g for 5 min at 4°C. Nuclei are washed once with PBS and resuspended in NT-2 Buffer (150mM NaCl), and sonicated (17–20%, 3×6 sec). Cells membranes and insoluble material is precipitated by centrifugation, 12000g for 10 min at 4°C. Supernatant is collected and protein concentration is measured with BCA assay. For each mg of extract, 33ul of Dynabeads G are incubated with 10μg antibody 20min at RT, washed 5times in NT-2 150 and incubated with 1mg of extract overnight at 4°C. IPs were washed one time with NT-2 buffer supplemented with 1 mg/ml Heparin and five times with 1 ml of NT-2 buffer (with 300mM NaCl). Proteins were analyzed by western blot.

### Immunofluorescence and laser-scanning confocal microscopy

U2OS cells were grown on 4 well glass slides (Millicell EZ SLIDE 4 well glass, Millipore Sigma PEZGS0416) with 5×10^5^ cells per wells for two days. After washing three times in 1× PBS (pH 7.4), cells were crosslinked in prechilled 2%PFA at 20°C for 15 min. After fixation, slides were washed three times in ice cold PBS and permeabilized in PBS + 0.2% Triton X-100 for 5 min at 20°C and blocked for 1h in Blocking solution (10% FBS and 0.1% Tween 20 in PBS). eIF4E antibody (SantaCruz A-10) was incubated overnight at 4°C in Blocking solution (1:100). After washing three times in PBS, cells were incubated with SON, SF3B1, NCBP2 or POLR2-S2P antibodies for 2 hours at room temperature. Slides were washed three times and incubated with secondary antibody (rabbit anti-RRX and mouse AF555) After washing three times in PBS, cells were mounted in antifade mounting medium with DAPI (Vector Laboratories, H2000). Analysis was carried out using a laser-scanning confocal microscope (Leica TCS SP8 DLS, LightSheet), 63× oil objective and numerical aperture of 1. Channels were detected separately, with no cross-talk observed. Confocal micrographs represent single sections through the plane of the cell. Images were obtained from LAS-X software (Leica Microsystems) and displayed using Adobe Photoshop CS6 (Adobe).

For quantification of co-localization images were opened with Imaris and the background was determined using control images of cells labeled with rabbit and mouse IgGs, followed by incubation with secondary antibodies (anti-mouse RRX and anti-rabbit AF488). The threshold was set to 50 for both colors. For quantification, multi-cell images were cropped to obtain at least 20 individual nuclei per condition. A DAPI mask was applied to specifically quantify the signal within the nuclear region of the cells. A colocalization channel was generated, and statistics were extracted from it. Mander’s coefficient was used to assess the degree of co-localization between the two signals. The percent co-localization was based on the ROI volume colocalized.

### Data analysis and visualization

Analysis and visualization of the differential gene expression, alternate splicing, and motif position data was done in R version 4.4.2 using the *tidyverse* suite of packages for data manipulation, *biomaRt* and *BSgenome* to retrieve genomic data, *pheatmap* for making heatmaps, *eulerr* for making Euler diagrams, and *ggplot2* for all other plots. The web-based version of metascape was used (https://metascape.org/gp/index.html#/main/step1) for gene ontology analysis employing default settings and accessed in June-July 2025^72^. Analysis and visualization of qPCR was performed in PRISM 10.4.2.

## Supplementary Material

Supplement 1

Supplement 2

Supplement 3

Supplement 4

Supplement 5

Supplement 6

Supplement 7

Supplement 8

Supplement 9

Supplement 10

Supplement 11

Supplement 12

Supplement 13

Supplement 14

Supplement 15

Supplement 16

Supplement 17

Supplement 18
