# Supplementary material for: Nuclear RNA cap-chaperones eIF4E and NCBP2 govern distinct fates for 1000s of mRNAs uncovering an unexpected regulatory point in gene expression
Source: bioRxiv. 2025 Jul 31:2025.07.25.666897. Preprint. [Version 2] doi: 10.1101/2025.07.25.666897 (PMC12324239; doi:10.1101/2025.07.25.666897)
Supplement: Supplement 17 [file media-17.docx]

**Supplemental Tables**

**Table 1**. Gene Ontology analysis of genes differentially expressed genes following eIF4E overexpression

**Table 2**. Metascape analysis of top 3 protein-protein interactions of genes differentially expressed genes following eIF4E overexpression

**Table 3**. Differentially expressed genes following NCBP2 overexpression in U2OS cells

**Table 4**. Gene Ontology analysis of genes differentially expressed genes following NCBP2 overexpression

**Table 5**. Genes that are differentially expressed in both eIF4E and NCBP2 overexpression

**Table 6**. All significantly changing alternative splicing events following NCBP2 overexpression

**Table 7**. Significantly changing skipped exon and mutually exclusive exon events following NCBP2 overexpression

**Table 8**. Intron lengths surrounding skipped exon events following NCBP2 overexpression

**Table 9**. Gene Ontology analysis of genes alternatively spliced following NCBP2 overexpression

**Table 10**. Genes with significant alternative splicing events in both eIF4E and NCBP2 overexpression

**Table 11**. Gene Ontology analysis of genes with alternative splicing events in both eIF4E and NCBP2 overexpression

**Table 12**. Skipped exon and mutually exclusive exon events in both eIF4E and NCBP2 overexpression

**Table 13**: RBPs similar to recognition motifs similar to enriched motifs in introns surrounding skipped exon events

**Table 14**: Enriched motifs in introns surrounding skipped exon events

**Table 15**: Antibodies used in western blots

**Table 16:** Primers used for qPCR
