# Supplementary material for: Nuclear RNA cap-chaperones eIF4E and NCBP2 govern distinct fates for 1000s of mRNAs uncovering an unexpected regulatory point in gene expression
Source: bioRxiv. 2025 Jul 31:2025.07.25.666897. Preprint. [Version 2] doi: 10.1101/2025.07.25.666897 (PMC12324239; doi:10.1101/2025.07.25.666897)
Supplement: Supplement 18 [file NIHPP2025.07.25.666897v2-supplement-18.pdf]

bioRxiv preprint doi: https://doi.org/10.1101/2025.07.25.666897; this version posted July 31, 2025. The copyright holder for this preprint (which was not certified by peer review) is the author/funder, who has granted bioRxiv a license to display the preprint in perpetuity. It is made available under aCC-BY 4.0 International license.

Figure S1

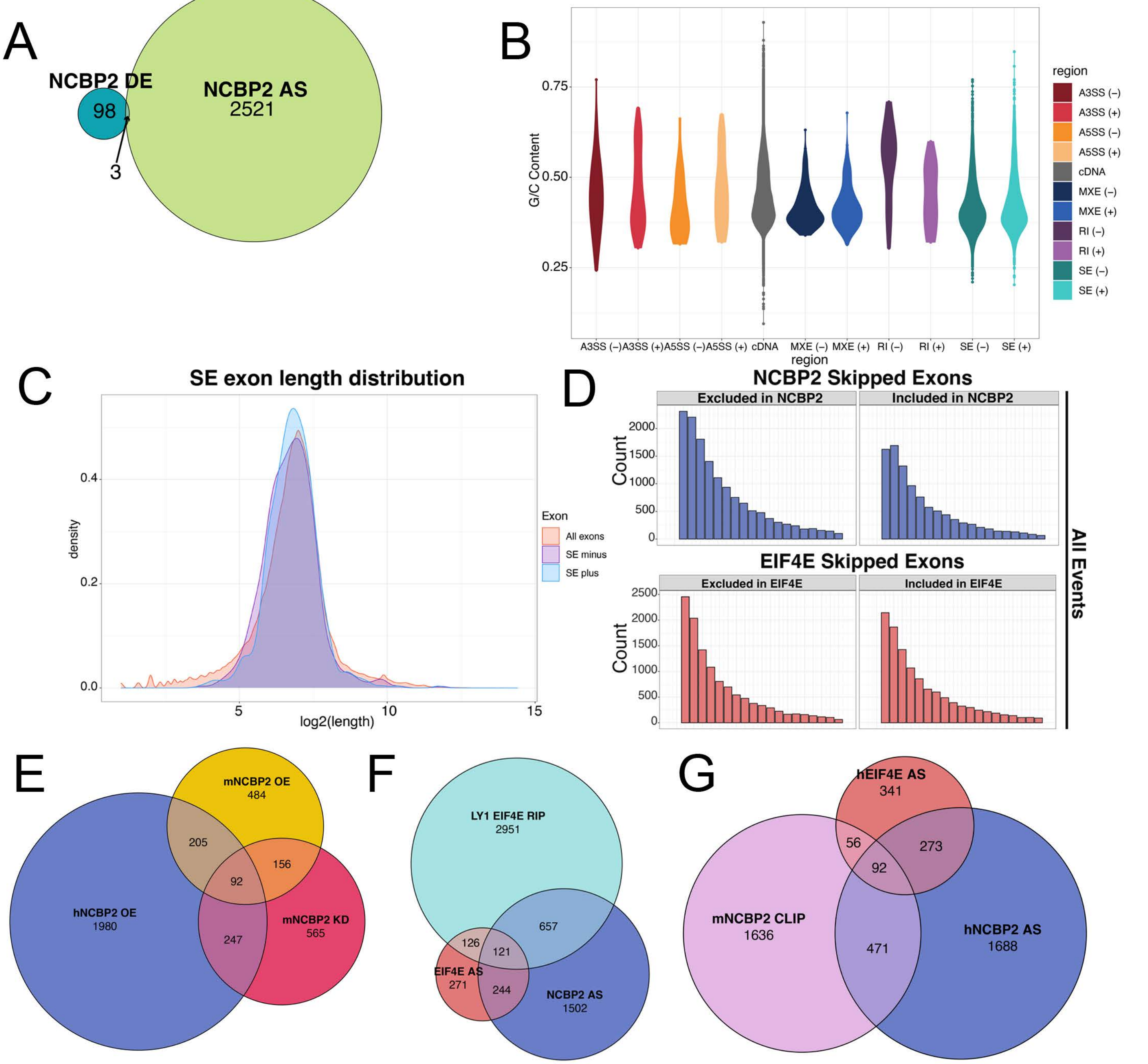

bioRxiv preprint doi: https://doi.org/10.1101/2025.07.25.666897; this version posted July 31, 2025. The copyright holder for this preprint (which was not certified by peer review) is the author/funder, who has granted bioRxiv a license to display the preprint in perpetuity. It is made available under aCC-BY 4.0 International license.

Figure S1: eIF4E and NCBP2 splicing targets have shared characteristics.

A) Euler plot showing the overlap between genes undergoing differential expression (blue) and alternative splicing (green) following NCBP2 overexpression. **B)** Violin plot showing the GC content of transcripts with alternate 3' splice sites (red), alternate 5' splice sites (orange), mutually exclusive exons (blue), retained introns (purple), skipped exons (teal), or all transcripts (grey) in NCBP2 overexpression. Lighter shades indicates events with more inclusion in NCBP2 (+). **C)** Density plot showing the length of all exons (red) or skipped exons that are more (blue) or less (purple) included in NCBP2 overexpression. **D)** Position of skipped exons in following overexpression of NCBP2 (blue, top) or eIF4E (red, bottom). **E)** Euler plot showing the overlap between genes undergoing alternative splicing as a result of NCBP2 overexpression in U2OS cells (blue), NCBP2 overexpression in mouse cardiomyocyte cells (yellow), or NCBP2 knockdown in mouse cardiomyocyte cells (pink). **F)** Euler plot showing the overlap between eIF4E RIP targets in LY1 cells (teal) and genes undergoing alternative splicing as a result of eIF4E (red) or NCBP2 (blue) overexpression. **G)** Euler plot showing the overlap between NCBP2 CLIP targets in mouse cardiomyocyte (pink) and genes undergoing alternative splicing in U2OS cells following overexpression of eIF4E (red) or NCBP2 (blue).

bioRxiv preprint doi: https://doi.org/10.1101/2025.07.25.666897; this version posted July 31, 2025. The copyright holder for this preprint (which was not certified by peer review) is the author/funder, who has granted bioRxiv a license to display the preprint in perpetuity. It is made available under aCC-BY 4.0 International license.

Figure S2

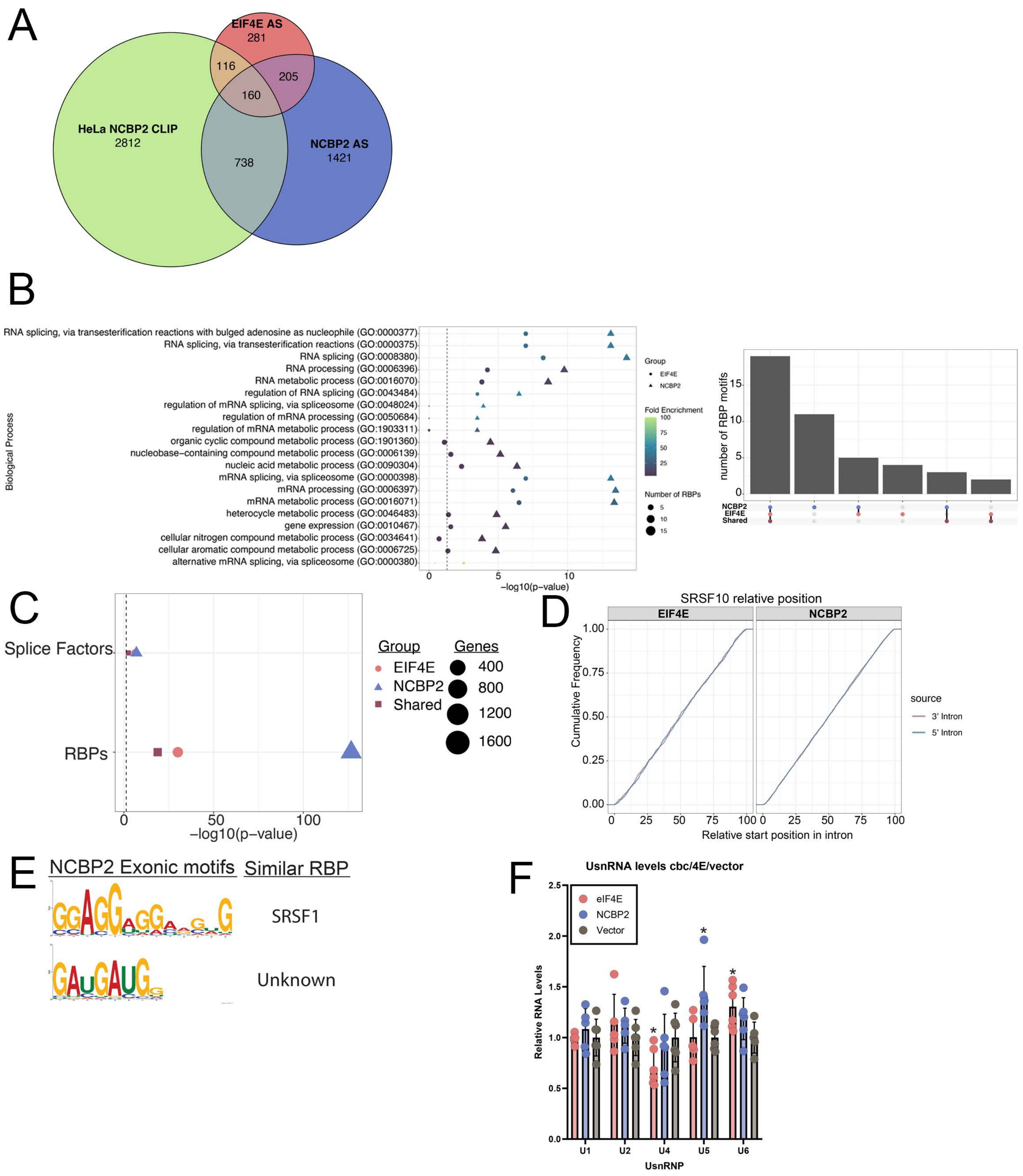

bioRxiv preprint doi: https://doi.org/10.1101/2025.07.25.666897; this version posted July 31, 2025. The copyright holder for this preprint (which was not certified by peer review) is the author/funder, who has granted bioRxiv a license to display the preprint in perpetuity. It is made available under aCC-BY 4.0 International license.

Figure S2: Motifs in eIF4E and NCBP2 splice targets are similar to known RBP motifs.

A) Euler plot showing the overlap between genes that are iCLIP targets of NCBP2 in HeLa cells (green) or undergo alternative splicing in U2OS following overexpression of eIF4E (red) or NCBP2 (blue). **B)** *Left* Dotplot showing the biological processes of RNA binding proteins that recognize motifs similar to sequences enriched in introns in eIF4E (circles), NCBP2 (triangles) skipped exon alternative splicing targets. Dashed line indicates a p-value of 0.05. *Right* Upset plot indicating the number of RNA binding protein with recognition motifs similar the introns surrounding skipped exons in NCBP2, eIF4E, or both. **C)** Dotplot showing enrichment of RNA binding proteins and splice factors in alternatively spliced genes. Dashed line indicates a p-value of 0.05. **D)** Relative position of the SRSF10 recognition motif in the upstream (blue) and downstream (pink) introns of genes undergoing alternative splicing in eIF4E (left) and NCBP2 (right). **E)** Motifs that were enriched in the sequences of exons skipped only as a result of NCBP2 overexpression. **F)** RT-qPCR analysis of UsnRNA levels in U2OS cells overexpressing eIF4E or NCBP2. Standard deviations and p-values relative to vector (Welch t-test) were calculated in PRISM. * = p-value < 0.05, all others were non-significant.
